# Association of Patient and Geographic Variables in Pediatric Patients With Prediabetes Becoming Lost to Follow-up

**DOI:** 10.1210/jendso/bvae157

**Published:** 2024-09-06

**Authors:** Maya Hamaker, Jessica Schmitt

**Affiliations:** Touro College of the Osteopathic Medicine, New York, NY 10027, USA; University of Alabama at Birmingham Diabetes Research Center, Birmingham, AL 35294, USA; Department of Pediatrics, University of Alabama at Birmingham, Birmingham, AL 35294, USA

**Keywords:** prediabetes, pediatrics, telemedicine, diabetes prevention

## Abstract

**Introduction:**

Prediabetes (PD) is becoming more common, and management is complicated by high rates of loss to follow-up. We evaluated variables associated with lost to follow-up status for pediatric patients with PD referred to endocrinology for evaluation and management.

**Methods:**

We evaluated new patients referred to Children's of Alabama Endocrinology for PD from March 2017 through March 2021. Variables included patient medical and demographics as well as county-level metrics. Comparisons of patients who returned to clinic and those who were lost to follow-up were assessed by chi-square for categorical variables and Student’s *t*-test/Wilcoxon rank sum test for continuous normal/skewed variables, respectively. Univariate logistic regression modeling identified risk factors for coming lost to follow-up and odds ratios with 95% confidence intervals were reported with a 2-sided *P*-value for significance of <.05.

**Results:**

A total of 524 patients were included in the analysis. Almost one-fourth of patients were lost to follow-up (24.6%). The odds of returning to clinic were higher in patients with the Children's Health Insurance Plan, who were prescribed endocrine medications, who had a concurrent diagnosis of cholesterol disorder, who had referral to the endocrine clinic before COVID-19, and who were offered a telehealth visit. No other assessed variable was significantly associated with the likelihood of returning to clinic.

**Conclusion:**

Independent of obesity severity, age, sex, race, county-level health, and economic variables, the factor most strongly associated with returning to clinic was having a telemedicine visit scheduled. Our data suggest that offering telemedicine visits may reduce lost to follow-up rates in this patient population.

Prediabetes (PD) is a serious medical condition that increases the risk of developing type 2 diabetes (T2DM), heart disease, and stroke. The Centers for Disease Control and Prevention reports PD affects 1 in 3 adults and up to 1 in 5 adolescents [1-3]. As progression from PD to T2DM can be delayed with lifestyle changes, it is important to diagnose and educate youth with PD as early as possible.

Often, pediatric primary care providers refer to specialty pediatric endocrine clinics for further management of PD and obesity. Loss to follow-up is a challenge for many specialty clinics, including endocrinology and clinics specializing in obesity [4-7]. One-third of pediatric patients with obesity seen in specific pediatric weight management programs do not follow-up within 1 year [7], and follow-up continues to be problematic in youth with T2DM. One study found 55% of pediatric patients with T2DM were lost to follow-up after 1.3 years [6]. As PD increases the risk of developing T2DM, special attention should be paid to barriers to follow-up for pediatric patients with PD.

Children's of Alabama is the only stand-alone children's hospital in Alabama and accepts referrals for PD across the state. Children's of Alabama accepts all insurances, including private insurance, Medicaid, and the Children's Health Insurance Plan (CHIP). Medicaid and CHIP insurance eligibility is determined by family income. To qualify for Medicaid, a family in Alabama's income must be at or below 146% of the federal poverty limit ($45 552 yearly for a family of 4 in 2024). To qualify for CHIP, a family in Alabama must have an income between 146% and 300% of the federal poverty level ($45 552-$93 600 yearly for a family of 4 in 2024). Telemedicine was offered at Children's of Alabama beginning in 2020 at the provider's discretion. While the medical doctor was present during scheduled telemedicine visits, dieticians were not. Providers could request dieticians contact families after the telemedicine consult visit, but this was at the providers’ discretion and was not uniform among all providers. Across the country, telemedicine visits were covered by all insurances following the onset of the coronavirus 2019 pandemic. As such, this clinic is uniquely positioned to assess characteristics of patients with PD who are lost to follow-up across a wide geographic area.

We aimed to determine whether patient-specific or geographic-dependent variables are more strongly associated with the likelihood of patients becoming lost to follow-up. We hypothesized that those with concurrent endocrine diagnoses such as hyperlipidemia, hypertriglyceridemia, or polycystic ovarian syndrome (PCOS) and those prescribed endocrine-related medications would be more likely to return to clinic.

## Methods

In this institutional review board-approved diabetes registry, the electronic medical record at Children's of Alabama was used to identify patients who were scheduled for a new outpatient consultation at the Pediatric Endocrine and Diabetes Outpatient Clinic for PD from March 1, 2017, through March 31, 2021. Diagnosis of PD was determined by provider entry of International Classification of Diseases, Tenth Revision (ICD-10) codes (R73.03). Those with a concurrent code for T2DM (E11.xx) were excluded. The diagnosis of PD was based on provider entry of ICD-10 codes entered at the time of the new patient consultation. While diagnosis of PD can be made with hemoglobin A1c, oral glucose tolerance test, or fasting glucose, the general procedure in this clinic is to utilize hemoglobin A1c for most patients. The primary outcome was whether patients returned to clinic or were lost to follow-up. Lost to follow-up was defined as not attending a second visit out of up to 4 scheduled visits. This was done to mitigate misappropriately identifying patients as returning to clinic when their appointment might have been cancelled or rescheduled due to the COVID-19 pandemic or provider schedule changes. The before and after COVID timeframe was defined as pre- or post-April 1, 2020, when Alabama stay-at-home orders went into effect [8].

### Exclusion Criteria

Patients with no insurance data and those who did not live in Alabama were excluded from the analysis. Additionally, new patients who were not scheduled for any follow-up visits were excluded.

### Covariates

Demographic data was collected from the patients' electronic medical records including sex, age, race/ethnicity, and insurance status. Race/ethnicity categories were defined as Hispanic, non-Hispanic White, non-Hispanic Black, and other by self-report. Insurance statuses were combined into 3 categories: private, Medicaid, and CHIP.

Clinical data analyzed included first visit or referral hemoglobin A1C, body mass index (BMI), current medications, and concurrent diagnoses. Hemoglobin A1C was determined at first visit or was acquired from referral paperwork if drawn within 3 months of the patient's first visit in the clinic. For analysis, the hemoglobin A1c at the time of visit was used when available, but the hemoglobin A1c at the time of referral may have differed. Current medications analyzed included metformin, statins, and glucagon-like peptide 1 (GLP-1) receptor agonists. Concurrent diagnoses analyzed included PCOS and cholesterol disorders determined by provider entry of ICD-10 codes E28.x and E78.x, respectively. Telehealth visits were offered at the discretion of the treating physician.

Geographic variables were obtained from the US Department of Agriculture Environmental Food Atlas and its primary data sources [9]. County-level variables included percentage of housing units with low access to a grocery store and no car, percentage of students eligible for the free lunch program, adult diabetes rate, child poverty rate, median household income, and driving time to the clinic. Data on car and grocery-store access was calculated from stores authorized to accept SNAP benefits, the Trade Dimensions' TDLinx directory of stores in the United States [10], and the household data from the 2010-2014 American Community Survey [11]. The percentage of students eligible to participate in the National School Lunch Program was collected from the US Department of Education, National Center for Education Statistics, Common Core of Data [12]. The adult diabetes mellitus rate (excluding gestational diabetes) in 2013 was collected from the Center for Disease Control and Prevention's Division of Diabetes Translation website [13]. The child poverty rate and median household income data were collected from the US Department of Commerce, Bureau of the Census, Small Area Income and Poverty Estimates [14]. Driving distance to Children's of Alabama was estimated by using Google Maps [15] using the clinic address and the patient's zip-code. The driving times were all calculated in between 9 and 10 Am to capture normal clinic times but avoid rush hour. To better estimate the impact of location on the patient, driving distance analysis was based on the lowest reported driving time for a personal vehicle.

### Statistical Analysis

Normality of continuous variables was determined with the Anderson–Darling normality test. Normal continuous variables were summarized by means and SD, while skewed variables were summarized by median and interquartile ranges (IQR). Categorical variables were summarized by counts and percentages. Comparisons of patients who returned to clinic and those who were lost to follow-up were assessed by chi-square for categorical variables and Student’s *t*-test/Wilcoxon rank sum test for continuous normal/skewed variables, respectively. We used a univariate logistic regression model to identify significant risk factors for lost to follow-up. Odds ratios (ORs) and 95% confidence intervals (CIs) were reported. Statistical analysis was performed with SAS® 9.4 software (SAS Institute, Cary, NC, USA). All tests were 2-sided, and significance was set at *P* < .05 for all comparisons.

## Results

### Subjects

**Figure 1.**
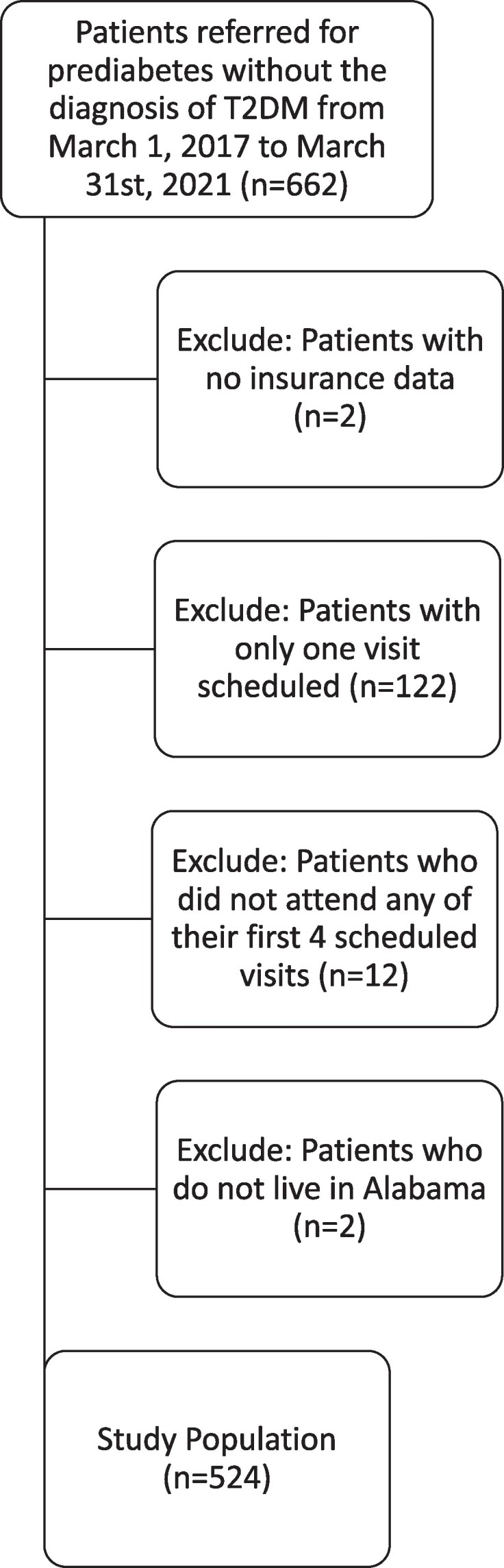
Study sample flow diagram.

From March 1, 2017, through March 31, 2021, 662 new patients were seen at Children's of Alabama Pediatric Endocrinology for PD. Of these patients, 2 were excluded due to lack of insurance data, and 122 were excluded because after initial consultation with pediatric endocrinology, they were not scheduled for follow-up with endocrinology based on provider evaluation at their visit. Twelve patients were excluded as they did not attend any of their first 4 scheduled visits, and an additional 2 patients were excluded as they did not live in Alabama. A total of 524 patients remained and were included in this analysis (shown in Fig. 1).

Overall, almost a fourth of patients were lost to follow-up (n = 129, 24.6%). The median age of patients was 12.8 (IQR: 10.0-15.1) years. Patients were predominantly female (n = 322, 61.5%) and non-Hispanic Black (n = 311, 59.4%). The proportion of patients with CHIP, Medicaid, and private insurance was similar (34.4%, 34.5%, and 31.1%). Overall, median number of visits scheduled was 3 (IQR: 2-4). Patients who returned to clinic had a median number of visits scheduled of 4 (IQR: 3-5) while those who were returning to clinic had a median schedule of 2 (IQR: 2-3) (*P* < .0001). A total of 350 initial visits occurred prior to COVID-19 with 174 occurring after. For those patients with an initial visit before COVID-19, 71 of them were lost to follow-up (20.3%). For patients with an initial visit after COVID-19, 58 of them (33.3%) were lost to follow-up (*P* = .0011) (shown in Table 1). Most patients did not have a telemedicine appointment scheduled (n = 430, 82.1%). Of the 94 patients who had a telemedicine appointment scheduled, 73 (77.7%) of them had their initial consultation before the COVID-19 pandemic.

**Table 1.** Demographics

	Total (n = 524)	Lost (n = 129, 24.62%)	Return (n = 395, 75.38%)	*P*-value
Age	12.8 (10.0-15.1)	13.3 (10.5-15.3)	12.8 (9.9-15.1)	.24
Sex				.88
Female	322 (61.5)	80 (62.0)	242 (61.3)	
Race/ethnicity				.37
Hispanic	53 (10.1)	9 (7.0)	44 (11.1)	
Non-Hispanic Black	311 (59.4)	78 (60.5)	233 (59.0)	
Non-Hispanic White	146 (27.9)	40 (31.0)	106 (26.8)	
Other	14 (2.7)	2 (1.6)	12 (3.0)	
Insurance				.0121
Chip	180 (34.4)	31 (24.0)	149 (37.7)	
Medicaid	181 (34.5)	55 (42.6)	126 (31.9)	
Private	163 (31.1)	43 (33.3)	120 (30.4)	
Number of scheduled visits	3 (2-5)	2 (2-3)	4 (3-5)	<.0001
COVID—time of initial visit				.0011
Before	350 (66.8)	71 (55.0)	279 (70.6)	
After	174 (33.2)	58 (45.0)	116 (29.4)	
Telehealth visit scheduled				.0005
Yes	94 (17.9)	10 (7.8)	84 (21.3)	

### Clinic Variables

Median hemoglobin A1C was 5.8 (IQR: 5.6-6.0), and median BMI was 32.8 (IQR: 27.2-38.6) at time of first visit. A minority (15.7%, n = 82) of patients had a concurrent cholesterol disorder, and 9.6% of the female patients had a concurrent PCOS diagnosis (n = 31 of 322 females). Almost half of the patients were on endocrine medications (n = 242, 46.2%). Of the patients on endocrine medications, the most common medication was metformin (n = 235, 97.1%) (shown in Table 2).

**Table 2.** Study sample clinical data

	Total (n = 524)	Lost (n = 129)	Return (n = 395)	*P*-value
Hemoglobin A1C*^a^*	5.8 (5.6-6.0)	5.8 (5.5-5.9)	5.8 (5.6-6.0)	.34
BMI*^b^*	32.8 (27.2-38.6)	34.2 (28.7-38.7)	32.4 (26.9-38.6)	.23
BMI Z-score*^c^*	2.4 (2.0-2.6)	2.4 (2.1-2.6)	2.4 (2.0-2.6)	.42
Diagnosed with cholesterol disorder				.0449
Yes	82 (15.7)	13 (10.1)	69 (17.5)	
Females diagnosed with PCOS*^d^*				.24
Yes	31 (9.6)	5 (6.3)	26 (10.7)	
Prescribed endocrine medication*^e^*				.0015
Yes	242 (46.2)	44 (34.1)	198 (50.1)	
Prescribed metformin				.0025
Yes	235 (44.9)	43 (33.3)	192 (48.6)	
Prescribed statin				.21
Yes	17 (3.2)	2 (1.6)	15 (3.8)	
Prescribed GLP-1 receptor agonist				.32
Yes	3 (0.6)	0 (0.0)	3 (0.8)	

All variables are expressed as n (%) and median (interquartile range).

Abbreviations: BMI, body mass index; GLP-1, glucagon-like peptide 1; PCOS, polycystic ovarian syndrome.

^*a*^Total (n = 488), Lost (n = 126), Return (n = 362).

^*b*^Total (n = 460), Lost (n = 120), Return (n = 340).

^*c*^Total (n = 400), Lost (n = 106), Return (n = 294).

^*d*^Total (n = 322), Lost (n = 80), Return (n = 242).

^*e*^Statin, GLP-1 receptor agonist, or metformin.

### County-level Variables

County-specific variables were similar in patients who returned to clinic and those who were lost to follow-up. While median household income varied slightly between those who returned to clinic and those who were lost to follow-up ($48 415 and $47 234, respectively, *P* = .17), no significant difference was seen between those who returned to clinic and those lost to follow-up in any county-specific variable, including driving time to the clinic (shown in Table 3).

**Table 3.** Study sample geographic-dependent data

	Total (n = 524)	Lost (n = 129)	Return (n = 395)	*P*-value
% housing units without a car and >1 mile from a supermarket	2.7 (2.6-3.2)	2.7 (2.7-3.1)	2.7 (2.6-3.2)	.63
% children in county on free school lunches	42.6 (41.9-52.7)	42.6 (42.6-53.4)	42.6 (41.8-52.7)	.53
Adult type II diabetes rate	13.0 (13.0-14.2)	13.0 (13.0-14.2)	13.0 (12.8-14.1)	.30
Child poverty rate	26.2 (24.7-30.7)	26.2 (25.1-31.1)	26.2 (24.0-30.7)	.22
Median income	48 415 (42 091-48 415)	47 234 (42 091-48 415)	48 415 (42 091-48 415)	.17
Driving time to clinic (minutes)	60 (24-91)	65 (26-91)	65 (24-91)	.63

**Figure 2.**
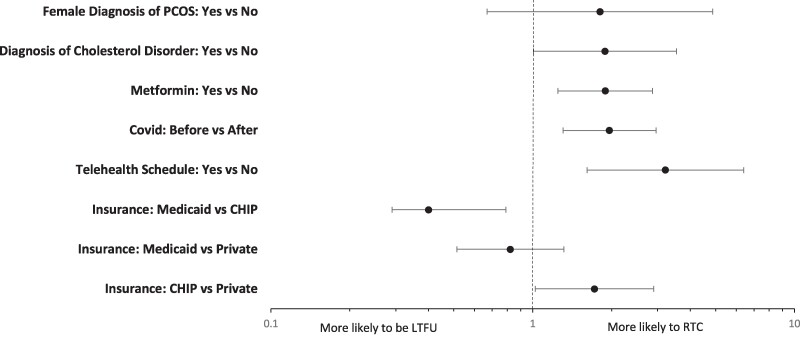
Odds of return to clinic. Significant increased odds of returning to clinic were seen by insurance status, prescribed endocrine medications, access to telehealth, and time of initial visit relative to COVID-19.

The odds of returning to clinic were higher in those with any scheduled telehealth visits, CHIP insurance, prescribed endocrine medications, prescribed metformin, concurrent diagnosis of cholesterol disorder, and referral to the endocrine clinic before COVID-19 stay-at-home orders went into effect (shown in Fig. 2). Sex, age, race/ethnicity, hemoglobin A1C, BMI, concurrent PCOS diagnosis, prescription of statins, prescription of GLP-1 receptor agonists, and all county-level variables were not associated with patients' odds of returning to clinic.

The odds of returning to clinic were higher for CHIP insurance compared to private insurance (OR 1.722, 95% CI 1.023-2.899). Those with Medicaid were less likely to return to clinic when compared to those with CHIP (OR 0.4, 95% CI 0.29-0.79, *P* = .0012). There was no significant association between those with Medicaid compared to private insurance (OR 0.821, 95% CI 0.513-1.315).

The odds of returning to clinic were higher in those visits scheduled before the COVID-19 pandemic (OR 1.965, 95% CI 1.305-2.959, *P* = .0012). Additionally, the odds of returning to clinic were higher for patients who had a telehealth appointment scheduled compared to those who did not (OR 3.213, 95% CI 1.614-6.399, *P* = .0009).

The odds of returning to clinic were higher for those who were prescribed any endocrine medication (OR 1.942, 95% CI 1.283-2.937, *P* = .0017) including metformin (OR 1.892, 95% CI 1.248-2.867, *P* = .0025). Odds of returning to clinic were also higher in those with a concurrent diagnosis of a cholesterol disorder (OR 1.889, 95% CI 1.007-3.544, *P* = .0477). All other variables examined, including concurrent diagnosis of females with PCOS (OR 1.81, 95% CI 0.67-4.87, *P* = .2433), were not associated with increased odds of returning to clinic.

## Discussion

Retention in the clinic is challenging, particularly in children affected by obesity. Among a large cohort of pediatric patients referred for evaluation and management of PD, we found that no county-level variables, including driving time to the clinic, were associated with the odds of returning to clinic. We did find higher retention in patients with any scheduled telehealth visits, CHIP insurance, a prescription for an endocrine medication, or a diagnosis of cholesterol disorder.

We predicted that patients being prescribed endocrine medications would have an increased likelihood of returning to clinic as being prescribed medications tends to warrant closer follow-up by providers. We were correct in this hypothesis, but when analyzed by specific medication, only metformin was associated with returning to clinic (Table 2) while statins and GLP-1 receptor agonists were not. While an interesting finding, the small sample size limits generalizability. There were 235 patients who were prescribed metformin; however, only 17 patients were prescribed a statin and 3 prescribed a GLP-1 receptor agonist.

The small proportion of patients prescribed a GLP-1 receptor agonist is best explained by the time of study and diagnoses of exclusion. GLP-1 receptor agonists were not approved by the Food and Drug Administration (FDA) for use in pediatric patients without T2DM until the end of 2020 [16, 17]. While FDA approval for children with obesity occurred in December 2020, insurance in Alabama still does not routinely cover these medications without concurrent T2DM, a diagnosis that would have excluded inclusion in this analysis. While we hypothesized that having any concurrent endocrine diagnosis in addition to PD would decrease the risk of being lost to follow-up, only those with concurrent cholesterol disorders, and not PCOS, had a higher chance of returning to clinic.

Follow-up rates have previously been found to be similar between public and private insurances, and Shoemaker et al found that insurance type was not associated with clinic loss to follow-up in pediatric patients with T2DM [6, 7]. However, our study found that patients with CHIP insurance were more likely than those with private insurance or Medicaid to return to clinic. Further research is needed to explore this difference and the factors of these insurances that could play a role in returning to clinic.

Our data showed that those with a scheduled telehealth visit had the greatest odds of returning to clinic. The increased odds of returning to clinic with telehealth-scheduled visits may be due to the increased convenience provided to patients, especially those who live far distances from their clinic. Additionally, a large proportion of patients who were offered telemedicine appointments (77.7% of those scheduled) had their initial consultation prior to the COVID-19 pandemic. In the early stages of COVID-19, when a large proportion of our community was very cautious about returning to city centers such as Birmingham, Alabama, patients may have been more inclined to follow up via virtual options. As Children's of Alabama receives referrals from a large geographic area, continuing to offer telehealth may play a role in reducing return to clinic rates in youth with PD; however, its impact outside of COVID-19 needs further evaluation to better understand its utility. While our data suggests that those who are offered telemedicine visits are more likely to attend their follow-up visits, data on whether they have similar outcomes to those seen in person is not available. Further evaluation of telemedicine's effect on not just retention in the clinic but also metabolic outcomes is essential and will require longer-term studies.

There are several limitations to this study. First is the retrospective nature of the study; inclusion for analysis was dependent on correct diagnosis coding by referrals and providers. The incorrect application or missed application of the PD code would result in an inaccurate study population. Second, the COVID-19 pandemic occurred during the time frame of this study. Stay-at-home orders, social distancing, closures, and general uncertainty surrounding the pandemic may have led to frequent rescheduling and canceling of visits, introducing a potential cofactor. Patients who initially scheduled a follow-up visit at Children's of Alabama but then decided to follow up elsewhere and canceled or no-showed their follow-up visit would be incorrectly classified as lost to follow-up in our study. Fourth, the number of patients in a few of our variable categories (prescribed GLP-1 receptor agonists, prescribed statins, and diagnosis of PCOS) was very small. The small number of patients in those samples may limit the generalization of those findings. In relation, GLP-1 receptor agonists were not approved by the FDA to be prescribed to pediatric patients for the majority of our study (liraglutide was approved in July 2019 for children with T2DM and for children with obesity in December 2020). As more medications for pediatric patients affected by obesity become available, understanding the relationship between their use and clinic retention will be an area of further study. Telehealth visits were not offered prior to the pandemic and after the pandemic may not have been equally accessible for all patients or utilized equally by all providers. Provider and patient preferences, along with technology availability and access, could have led to varying telehealth access in this study. Finally, patients seen in-person at Children's of Alabama Endocrinology, have access to same-day in-person nutrition consultations. While standard operating procedures for those with new-onset PD receiving a consultation in this clinic is to have an in-person same-day dietician consultation, we were unable to include this in our variables and therefore cannot comment on if receiving formal dietary counseling from a registered dietician was associated with increased odds of return to clinic.

## Conclusions

We set out to determine if variables intrinsic to the patient (age, sex, race/ethnicity, insurance status, etc.) or those dependent on their county were most associated with being lost to follow-up. We found that in this cohort of youth with PD, the factor most strongly associated with returning to clinic was having telemedicine visits scheduled. Further work is needed to study the effects of telemedicine in the management of PD and best practices regarding nutrition and exercise counseling via telemedicine. Our work suggests, however, that telemedicine may play an important role in the management of pediatric patients with PD.

## Data Availability

Data available upon reasonable request and approval from the University of Alabama at Birmingham institutional review board.
